# Investigating insecticide susceptibility status of adult mosquitoes against some class of insecticides in Osogbo metropolis, Osun State, Nigeria

**DOI:** 10.1371/journal.pone.0285605

**Published:** 2023-05-11

**Authors:** Lateef. O. Busari, Haleemah O. Raheem, Zarat O. Iwalewa, Kamilu A. Fasasi, Monsuru A. Adeleke

**Affiliations:** 1 Faculty of Basic and Applied Sciences, Department of Zoology, Parasitology and Vector Biology Unit, Osun State University, Osogbo, Osun State, Nigeria; 2 Faculty of Basic and Applied Sciences, Department of Zoology, Applied Entomology and Pest Management Unit, Osun State University, Osogbo, Osun State, Nigeria; Federal University of Agriculture, Abeokuta, NIGERIA

## Abstract

The study evaluates the resistance and susceptibility of adult female *Anopheles gambiae* s. l., *Aedes aegypti* and *Culex quinquefasciatus* mosquitoes sourced within Osogbo metropolis, Osun State, Nigeria to four groups of insecticides [Permethrin, Deltamethrin, Pirimiphos-methyl and DDT (Dichlorodiphenyltrichloroethane)] and the distribution of their larval habitat within the metropolis. Mosquito larvae of the three genera were collected during the wet season and reared to adult stage in the laboratory. Emerged adult female mosquitoes were exposed to insecticide impregnated papers of the four insecticide groups for 60mins using WHO kits to determine the knock down rate (kdr). Thereafter, they were transferred into holding tubes and left for 24hrs to assess their resistance and susceptibility according to the WHO protocol. Four types of larval habitats were identified (tires, ground pools, gutters and plastic containers). *Anopheles gambiae* s. l. showed the highest resistance to Permethrin (49%) (p = 0.04, p<0.05) while the highest susceptibility was recorded with Pirimiphos-methyl (69%) with the lowest against Permethrin (16%) (P = 0.002; p<0.05). The highest resistance of *A*. *aegypti* was against OC-Control (45%) (p = 0.031; p<0.05). Permethrin had the highest susceptibility (60%) against *A*. *aegypti* while OC-control had the lowest (11%) (p = 0.005; p< 0.05). *Culex quinquefasciatus* had a lower resistance to OC-control (38%) as compared with *Aedes aegypti* (45%). However, it was least susceptible to Pirimiphos-methyl (52%) and DDT (17%) respectively (p = 0.013; p<0.05). The susceptibility of *A*. *gambiae* s. l. and *C*. *quinquefasciatus* to Pirimiphos-methyl and *A*. *aegypti* to Permethrin is an indication of the possibility of success if employed for vector control of *A*. *gambiae* s. l., *C*. *quinquefasciatus* and *A*. *aegypti* respectively. This could be through their inclusion as active ingredients in insecticide treated nets (ITNs) and indoor residual spray (IRS) with a view to abating malaria and other life-threatening mosquito-borne diseases constituting global public health scourge.

## Introduction

The public health menace of adult mosquitoes as vectors in the transmission of mosquito-borne diseases worldwide is worrisome. They transmit life-threatening diseases such as dengue fever, malaria, bancroftian filariasis, yellow fever, chikunguya fever, etc. [1]. Mosquitoes of the genera *A*. *gambiae* s. l., *A*. *aegypti* and *C*. *quinquefasciatus* are mostly vectors responsible for the transmission of the deadly mosquito-borne diseases in human causing nearly a million deaths and over 700 million infections globally annually [2]. Mosquitoes are cosmopolitan and are found in a variety of habitats including sewage water, stagnant water and fresh water [3]. *Anopheles gambiae* s. l. are the vectors of malaria accounting for about 214 million cases of malaria leading to 438,000 deaths annually globally [4]. About 1.1 billion people are at risk of contracting lymphatic filariasis [4], which is majorly transmitted by *C*. *quinquefasciatus* and species of *Anopheles* and *Mansonia*. *Culex tritaeniorhynchus* is the major vector of Japanese encephalitis, which is found in tropical and sub-tropical countries [5]. *Aedes aegypti* and *A*. *albopictus* mosquitoes are mainly responsible for transmitting dengue and dengue hemorrhagic fever, yellow fever, and chikungunya. It was reported that dengue infections affect about 2.4 million persons annually [4].

Despite global efforts in the prevention of mosquito-borne diseases through preventive chemotherapy and vector control programmes, the burden of the diseases persists through the emergence of resistant vectors to insecticides used for vector control. Although, despite the six classes of insecticides namely, Organochlorines, Organophosphates, Carbamates, Pyrethroids, Pyrroles and Phenyl-pyrazoles insecticides are currently used in mosquito control programmes worldwide [4], resistance has still been extensively reported. Thus, the necessity for rapt understanding of the resistant mechanism adopted by mosquitoes with a view to making broad spectrum insecticides that will be employed in vector control programmes.

Nigeria accounted for 25% of the 92% estimated malaria cases that occurred in Africa in 2017 where 217million malaria cases was reported [6] an indication of the endemicity of the country for malaria and other mosquito-borne diseases. The incidence of malaria, however, is reported to be increasing in Nigeria [7].

Invariably, Osun state is also endemic for mosquito-borne diseases [8]. Furthermore, the distribution of larval habitats and insecticide susceptibility status of *A*. *gambiae* complex is yet unknown in the State [9]. Thus, the need for the present study in assessing whether mosquitoes in Osogbo metropolis are resistant or susceptible to insecticides currently used for vector control of adult mosquitoes with a view to corroborating global efforts in the prevention and eradication of mosquito-borne diseases.

## Materials and methods

### Ethical approval

Ethical approval was sought and obtained from the health planning, research and statistics department, Osun State Ministry of Health, Abere, Osogbo, Osun State, Nigeria, to scout for mosquito larvae within Osogbo metropolis for mosquitoes’ breeding sites around human dwellings.

### Source of mosquito larval collection

The mosquito larvae used for this study were sourced within Osogbo metropolis. Osogbo is the capital city of Osun State, Nigeria. It is located on latitude (7° 49`N) and (7° 28`60`E) and at an elevation of 321 meters above the sea level in southwestern, Nigeria. The city experiences two seasons, the dry season (December to March) and wet season (April to October). The study was conducted between May and September 2022 during the wet season, which is associated with rainfall, thus the presence of breeding sites for the mosquitoes.

### Mosquito larval sampling and laboratory rearing to adult stages

Larva collection was done using scoops, dippers, containers and sieves of about 0.55mm mesh size into well labelled transparent containers netted to prevent escape of those that may emerge to adult during transportation to the laboratory. In the laboratory, they are reared to adult stages under standard conditions before the insecticide susceptibility test. Adult mosquito identification was done under a dissecting microscope using morphological keys described by [10–12]. Also, the types of breeding sites for mosquitoes were observed and noted.

### Bioassay

Emerged adult mosquitoes were tested for insecticide susceptibility/resistance using insecticide impregnated papers (4% DDT, 0.05% Deltamethrin, 0.75% Permethrin, Pirimiphos-methyl and OC- control) as described by the World Health Organization protocol [4]. About 25 adult female mosquitoes were aspirated into each of the four exposure tubes for WHO bioassay containing insecticide impregnated papers of each of the insecticides used in an upright position to make four replicates for each insecticide and monitored for an hour. At the end of an hour, moribund (knockdown) mosquitoes were transferred to holding tubes while the dead ones are removed. A paper without insecticide was used as the control.

In the holding tubes, the moribund adult female mosquitoes are left for 24hrs at end of which the dead mosquitoes i.e., susceptible, and those that are alive i.e., resistant, were counted, recorded preserved using silica gel in an Eppendorf tubes. This process was repeated for adult female *A*. *gambiae* s. l., *A*. *aegypti* and *C*. *quinquefasciatus* mosquitoes respectively.

### Data analysis

The data analysis was done using chi-square to test for the significant difference in insecticide resistance or susceptibility of the adult female mosquitoes to the insecticides used in the study, with a p-value of 0.05 indicating significance.

## Results

A total number of 1500 mosquito larvae were collected between May to September, 2022 in seven different locations within Osogbo metropolis. The mosquito larva were collected from different breeding sites and are identified to belong to the genera: *Anopheles*, *Culex* and *Aedes* (Table 1).

**Table 1:** Mosquito larval genera composition in different habitats in the study area.

Habitat Types	Habitat frequency	Mosquito larval genera present			Percentage composition
		*A*. *gambiae* s. l.	*C*. *quinquefasciatus*	*A*. *aegypti*	(%)
Gutter Ground pool Tyres Abandon plastics	4 10 4 2	3 9 0 0	4 6 0 0	0 0 4 2	14.29 35.31 14.29 7.14

After 60min of exposure, the highest kdr was observed with Pirimiphosmethy (85%) against *A*. *gambiae* s. l. while OC-control had lowest kdr (0%) against *A*. *aegypti* (p = 0.69, p>0.005). It also recorded the highest susceptibility (69%) against *A*. *gambiae* s. l. after 24hours of exposure while OC-control had the lowest mortality rate (11%) against *A*. *aegypti* (p = 0.65; p>0.05). Permethrin (49%) had highest resistance rate against *A*. *gambiae* s. l. over *C*. *quinquefasciatus* (24%) and *A*. *aegypti* (6%) while Pirimiphos-methyl showed the lowest resistance rate across the three mosquito genera [Table 2].

**Table 2:** kdr and mortality of the three mosquitos’ species after 60min and 24hrs exposures.

Insecticide	Knockdown rate after 60min (%)			Female Mortality rate after 24hours (%)			Female Resistance after 24hours (%)		
	*A*. *gambiae* s. l.	*C*. *quinquefasciatus*	*A*. *aegypti*						
				*A*. *gambiae* s. l.	*C*. *quinquefasciatus*	*A*. *aegypti*	*A*. *aegypti*	*C*. *quinquefasciatus*	*A*. *aegypti*
Permethrin	20	50	60	16	41	60	49	24	6
Deltamethrin	36	68	40	39	37	59	21	11	11
Pirimiphos-methyl	85	70	20	69	52	56	0	0	10
DDT	5	8	8	47	17	17	7	33	44
OC-control	1	0	0	26	15	11	20	38	45

**Fig 1:**
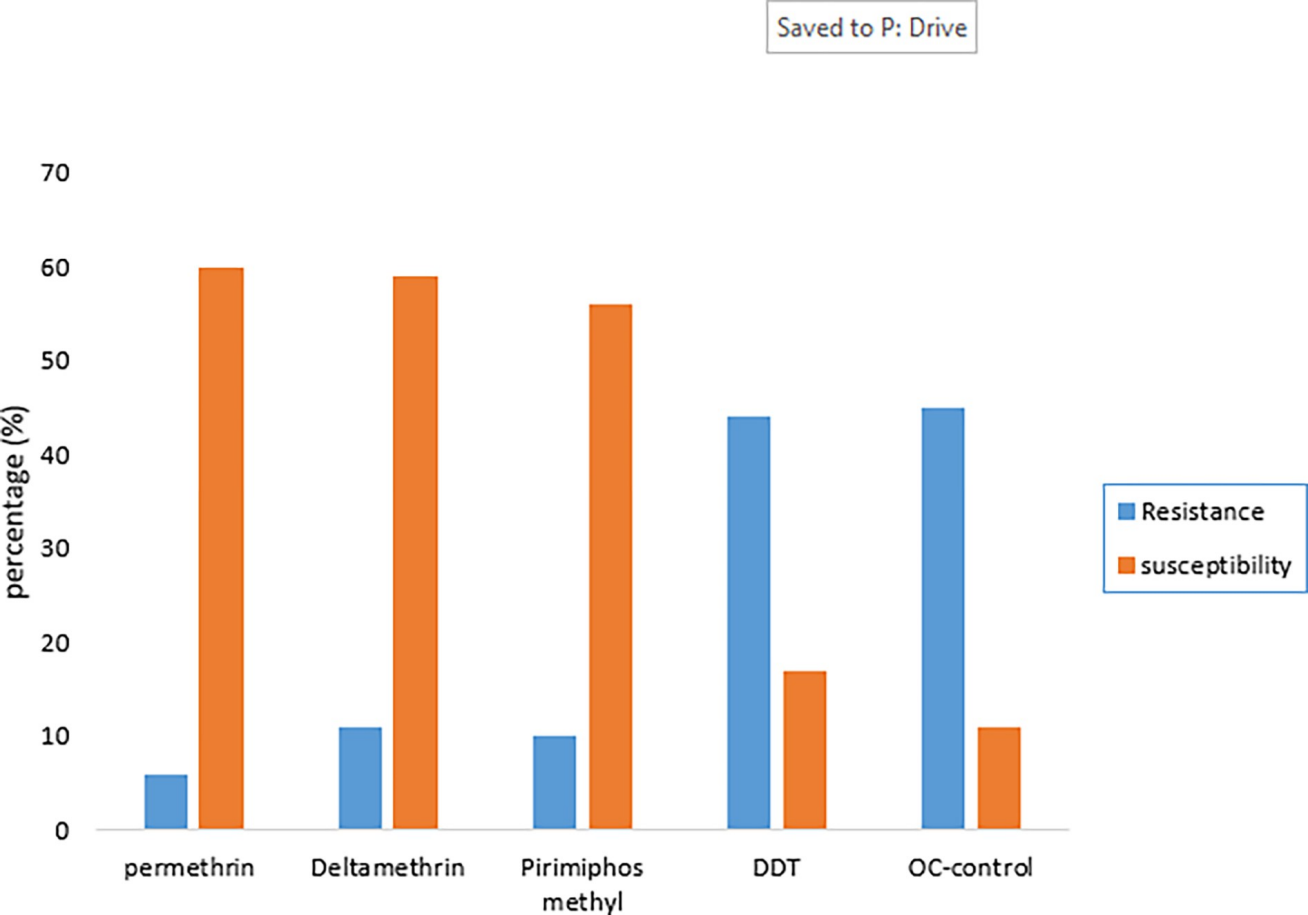
Resistance and susceptibility of *A*. *aegypti* after 24hrs exposure.

Against *A*. *aegypti*, Permethrin had the highest kdr (60%) with OC-control having the lowest (0) after 60mins of exposure [Table 3]. The *A*. *aegypti* had the highest resistance against OC-Control (45%)(p = 0.031; p< 0.05) [Fig 1]. Permethrin had the highest susceptibility (60%) against *A*. *aegypti* while OC-control had the lowest (11%) (p = 0.005; p< 0.05) [Fig 1].

**Table 3:** kdr for *A*. *aegypti* after exposure for 60minutes.

Insecticide (s)	Knockdown rate after 60min.
Permethrin	60
Deltamethrin	40
Pirimiphos-methyl	20
DDT	8
OC-control	0

**Fig 2:**
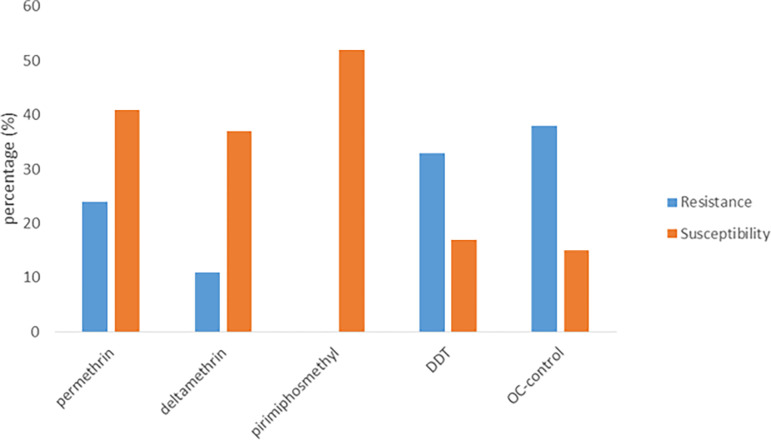
Resistance and susceptibility of *C*. *quinquefasciatus* after 24hrs exposure.

Pirimiphos-methyl had the highest kdr (70%) against *C*. *quinquefasciatus* while OC-control had the lowest (0%) after 60mins [Table 4]. It had the greatest resistance to OC-control (38%), DDT (33%), Permethrin (24%), Deltamethrin (11%) and Pirimiphos-methyl (0%) respectively (p = 0.016, p<0.05) (Fig 2). However, it was least susceptible to Pirimiphos-methyl (52%) and DDT (17%) respectively (p = 0.013; p<0.05) [Fig 2].

**Table 4:** kdr for *C*. *quinquefasciatus* after 60min exposure.

Insecticide(s)	Kdr
Permethrin	50
Deltamethrin	68
Pirimiphos-methyl	70
DDT	8
OC-control	0

**Fig 3:**
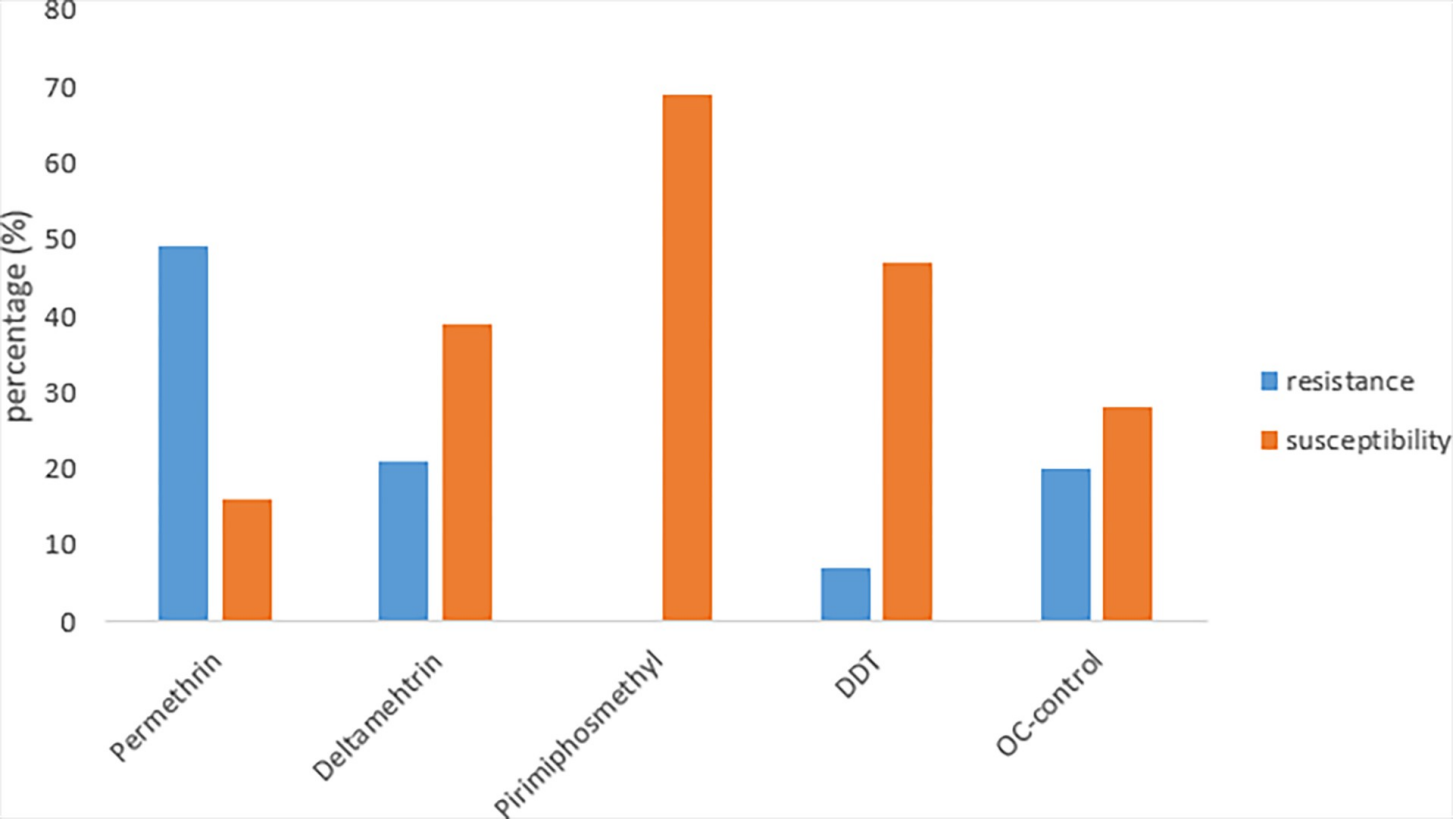
Resistance and susceptibility of *A*. *gambiae* s.l after 24hrs exposure.

After 60min exposure of *A*. *gambiae* s.l, Pirimiphosmethyl had the highest kdr (85%) with DDT having the lowest (5%) [Table 5]. The vector showed the greatest resistance to Permethrin (49%), Deltamethrin (21%), OC-control (20%), DDT (5%) and Pirimiphos-methyl (0%) respectively (p = 0.04, p<0.05) [Fig 3]. Furthermore, the highest susceptibility was recorded with Pirimiphos-methyl (69) while the lowest was with Permethrin (16) (P = 0.002; p<0.05) [Fig 3].

**Table 5:** kdr of *A*. *gambiae* s. l. after 60min exposure.

Insecticide(s)	Kdr
Permethrin	20
Deltamethrin	36
Pirimiphos-methyl	85
DDT	5
OC-control	1

## Discussion

Hitherto, the use of insecticides in the control of mosquito and eradication of mosquito-borne diseases has been largely employed while their efficiency determined through the knock down rate. The knock down rate of the vectors varied across the insecticides as the highest kdr was recorded in Pirimiphos-methyl against the three species after exposure (p = 0.04; p<0.05). This was followed by Permethrin (p = 0.02; p<0.05), Deltamethrin (p = 0.008; p>0.05), DDT and OC-control having the lowest kdr (p = 0.04; p<0.05) respectively. This portends the insecticidal efficacy of the insecticides in the impregnated papers against the three mosquito vector species as evident from their kdr. Furthermore, the assessment of the kdr showed that the tested insecticide impregnated paper induced knockdown of the adult female mosquito vectors, suggesting that knockdown mechanism could be present in the local mosquito populations in the study area. This conforms with earlier studies which indicated the knockdown effect of impregnated papers against the mosquito species in Nigeria [13–16].

After 24hrs exposure, *A*. *gambiae* s. l., *C*. *quinquefasciatus* and *A*. *aegypti* were resistance to OC-control (p = 0.009; p>0.05). This could be due to reported cases of vector resistance to organochlorine class of insecticides [17]. According to the WHO [1996], *Culex*, *Aedes* and *Anopheles* species have developed resistance rapidly to various insecticides in many countries, but *Culex* can develop resistance more rapidly to insecticides than other mosquitoes. The high resistance of *A*. *gambiae* s. l., *C*. *quinquefasciatus* and *A*. *aegypti* compared with OC-control and DDT (p = 0.0004; p<0.05), is in conformity with previous studies where emerging resistance to DDT have been reported [9]. Resistance was high but moderate in Permethrin (48%) and Deltamethrin (60%) (p = 0.002; p<0.05). The vectors showed a high susceptibility to Pirimiphos-methyl (70%) (p = 0.002; p<0.05) which is in consonance with findings by [18]. Furthermore, [19] reported that the mosquito species were susceptible to organophosphate but resistance to DDT with reduced susceptibility to Pyrethroid (Permethrin and Deltamethrin).

The low mortality rate with OC-control and DDT could be due to the presence of kdr mutation which is conferring resistance to the vectors since the spade of rapid resistance to insecticide by the vectors has been attributed to mutation. This could also be due to environmental factors [19] since environmental management is germane in vector control and disease elimination.

## Conclusion

Owing to the endemicity of mosquito-borne diseases such as malaria, lymphatic filariasis etc in the state and globally, the susceptibility of *A*. *gambiae* s. l. and *C*. *quinquefasciatus* to Pirimiphos-methyl and *A*. *aegypti* to Permethrin respectively. According to the present study, we suggest the possibility of success of vector control using Pirimiphos-methyl against *A*. *gambiae* s. l. and *C*. *quinquefasciatus* and Permethrin against *A*. *aegypti*. Thus, the need for the state government and health agencies to employ these insecticides in mosquito-borne diseases vector control programmes in the state.

## References

[1] Grigoraki L, Balabanidou V, Pipini D, Strati F J. Analysis of Insecticide Resistance in Mosquito Disease Vectors: From Molecular Mechanisms to Management. *Nova Acta Leopoldina* NF Nr. (2016) 411: 165–171.

[2] Caraballo H and King K. Emergency department management of mosquito-borne illness: malaria, dengue, and West Nile virus. *Emergency Medicine Practice* (2014), 16(5):1–23; quiz 23-4. . 25207355

[3] WHO (2013). Test procedures for insecticide resistance monitoring in malaria vector mosquitoes. World Health Organization; Geneva, Switzerland, 54pp. https://apps.who.int/iris/bitstream/handle/10665/250677/9789241511575-eng.pdf.

[4] WHO (2016). Test procedures for insecticide resistance monitoring in malaria vector mosquitoes, 2nd ed. Geneva: World Health Organization; 2016. https://apps.who.int/iris/bitstream/handle/10665/250677/9789241511575-eng.pdf?sequence=1 (Accessed 15 September 2022).

[5] CDC (Center of disease control) (2016). Surveillance and control of Aedes aegypti and Aedes albopictus in the United State. *Division of vector-borne disease*. PP:4–8.

[6] WHO (2017). World Malaria Report 2017. Geneva: World Health Organization; 2017. p. 31–40. https://www.who.int/malaria/publications/world-malaria-report-2017/en/. Page: 196. (Accessed 15 September 2022).

[7] WHO (2018). World Malaria Report 2018. Geneva: World Health Organization; 2018. p. 32–59. https://www.who.int/malaria/publications/world-malaria-report-2018/en/. Page: 210. (Accessed 15 September 2022).

[8] Adefioye O. A, Adeyeba O. A, Hassan W. O and Oyeniran O. A. Prevalence of malaria parasite infection among pregnant women in Osogbo, Southwest Nigeria. *American–Eurasian Journal of Scientific Research* (2007), 2(1): 43–45.

[9] Adeleke M. A, Adeyemi J. A, Fasasi K. A, Oforka L. C, Adeogun A. O, and Olatunde G.O. Molecular Characterization and Insecticide Susceptibility Status of *Anopheles gambiae* Complex (Giles, 1902) in Osun State, Southwestern Nigeria. *Nigeria Journal of Entomology* (2018), 34: 69–76. doi: 10.36108/NJE/8102/43 (0180).

[10] Hopkins G. H. E. Mosquitoes of the Ethiopian Region I.—Larval bionomics of mosquitoes and taxonomy of culicine larvae. 2nd ed. London: British Museum (1953) pp: 363.

[11] Gillet J. D. Common African mosquitoes and their medical importance (with colour illustrations). *W Heffer and Sons*, London (1972). pp: 120.

[12] Coetzee M. Keys to the females of Afrotropical Anopheles mosquitoes (Diptera: Culicidae). Malaria Journal (2020) 19(70): 1–20. 10.1186/s1236-020-3144-9. 32054502 PMC7020601

[13] Oduola A. O, Obansa J. B, Ashiegbu C. O, Adeogun A., Otubanjo O. A. and Awolola T. S. High level of DDT resistance in the malaria mosquito: *Anopheles gambiae s*. *l*. from rural, semi urban and urban communities in Nigeria. *Journal of Rural Tropical Public Health* (2010), 9: 114–120.

[14] Olayemi I. K, Ande A. T, Chita S, Ibemesi G, Ayanwale V. A and Odeyemi O. M. Insecticidal susceptibility profile of the principal malaria vector *Anopheles gambiae s*.*l*. (Diptera: Culicidae) in North central Nigeria. *Journal of Vector Borne Disease* (2011), 48(2):109–112. 21715735

[15] Umar A, Kabir G. B. J, Amajoh C. N, Inyama P. U, Ordu D. A, Barde A. A, et al. Susceptibility of female *Anopheles* mosquitoes to ten insecticides for indoor residual spraying (IRS) baseline data collection in North- Eastern Nigeria. *Journal of Entomology and Nematology* (2014), 6(7): 98–103.

[16] Awolola T. S, Adeogun A, Olakiigbe A. K, Oyeniyi T, Olukosi Y. A, Okoh H, et al. Pyrethroids resistance intensity and resistance mechanisms in *Anopheles gambiae* from malaria vector surveillance sites in Nigeria. *PLoS One* (2018). 10.1371/journal.pone.0205230. PMC6281219 30517090

[17] Adeogun A. O, Omotayo A, Babalola A, Joseph T, Adesalu O, Jimoh R, et al. High Pyrethroid Resistance to Deltamethrin and DDT in Major Malaria Vector *Anopheles gambiae* s. l. from South-Western Nigeria is Probably Driven by Metabolic Resistance Mechanisms. *Preprints* (2022), 2022060243. doi: 10.20944/preprints202206.0243.v1

[18] Corbel V, Guessan R. N, Brengues C, Chandre F, Djogbenou L, Martin T, et al. Multiple insecticide resistance mechanisms in *Anopheles gambiae* and *Culex quinquefasciatus* from Benin, West Africa. *Acta Tropica*, (2007) 101: 207–216. doi: 10.1016/j.actatropica.2007.01.005 17359927

[19] Nkya T. E, Akhouayri I, Poupardin R, Batengana B, Mosha F, Magesa S, et al. Insecticide resistance mechanisms associated with different environments in the malaria vector *Anopheles gambiae*: a case study in Tanzania. *Malaria Journal*, (2014) 13:28. doi: org/10.1186/1475-2875-13-28 24460952 10.1186/1475-2875-13-28 PMC3913622

